# A Fiber Bragg Grating Sensing Structure for the Design, Simulation and Stress Strain Monitoring of Human Puncture Surgery

**DOI:** 10.3390/s19143066

**Published:** 2019-07-11

**Authors:** Pengwen Xiong, Xin Huang, Yulong Li, Peter X. Liu

**Affiliations:** 1 School of Information Engineering, Nanchang University, Nanchang 330031, China; 2 School of Instrument Science and Engineering, Southeast University, Nanjing 210096, China; 3 Key Lab for Robot &Welding Automation of Jiangxi Province, School of Mechanical and Electrical Engineering, Nanchang University, Nanchang 330031, China; 4 Department of Systems and Computer Engineering, Carleton University, Ottawa, ON K1S 5B6, Canada

**Keywords:** puncture surgery, force sensing, fiber Bragg grating, spoke-type structure, new type elastic beam

## Abstract

In order to improve the precision and stability of puncture surgical operations to assist doctors in completing fine manipulation, a new of type puncturing needle sensor is proposed based on a fiber Bragg grating (FBG). Compared with the traditional puncture needle sensor, the new type of puncturing needle sensor is able to sense not only the axial force, but also the torque force during the puncture process. A spoke-type structure is designed near the needle tip. In order to eliminate the influence of temperature and realize temperature compensation, a reference fiber method using three FBGs is applied. FBG1 and the reference FBG2 are pasted on the upper and lower surfaces of the new-type elastic beam, and FBG3 is pasted into the groove on the surface of the new type of puncturing needle cylinder. The difference of Bragg wavelength between FBG1 and the reference FBG2 is calibrated with the torque force, while the difference between the Bragg wavelength of the FBG3 and the reference FBG2 is calibrated with the axial force. Through simulation and sensing tests, when the torque force calibration range is 10 mN·m, the torque average sensitivity is 22.8 pm/mN·m, and the determination coefficient R^2^ is 0.99992, with a hysteresis error Y_H_ and repetition error Y_R_ of 0.03%FS and 0.81%FS, respectively. When the axial force calibration rang is 5 N, the axial force average sensitivity is 0.089 nm/N, and the determination coefficient R^2^ is 0.9997, with hysteresis error Y_H_ and repetition error Y_R_ of 0.014%FS and 0.11%FS, respectively. The axial force resolution and torque resolution of the new type of puncturing needle sensor are 0.03 N and 0.8 mN·m, respectively. The experimental data and simulation analysis show that the proposed new type of puncturing needle sensor has good practicability and versatility.

## 1. Introduction

Minimally invasive surgery delivers surgical instruments to the human body and removes pathological tissue through a small wound [1,2]. Compared with traditional surgery, minimally invasive surgery has the advantages of small incisions, less pain, less bleeding and faster recovery [3]. For example, when extracting effusions in the thoracic cavity, if the lungs are compressed by the effusion for a long time, fibrin will easily organize and produce two layers of pleura adhesion, thereby affecting the breathing function of the lungs [4], so the effusion must be removed through puncture surgery. The slender and flexible surgical instruments [5] used in minimally invasive surgery, such as catheters and flexible needles, reduce the doctor’s perception of the interaction between distal end of the instrument and soft tissue, and make it difficult to achieve accurate manipulation of the instrument.

In minimally invasive surgery, doctors usually can only obtain the proximal operating force of the surgical instrument [6] but cannot obtain directly the contact force between the distal end of the surgical instrument and the tissue. The manual operation of doctors may be affected by physiological hand tremor, fatigue, patient movement and poor kinesthetic feedback. Therefore, the absence of force sensing is a formidable technical challenge. Applying excessive forces can cause damage to human health organs and tissues during puncture surgery, leading to potentially irreversible damage [7]. Thoracentesis refers to the operation of punctured into the pleura cavity through the skin, intercostal tissues, and parietal pleura by a disinfected puncture needle [8]. Therefore, tactile information [9,10,11,12] is an important factor affecting the safety of minimally invasive surgery, and tactile feedback could help to reduce tissue damage [13] caused by surgical instruments. Among the tactile information, force information is the most important features and need to accurately detect. According to the principle of force measurement, force sensors are divided into piezoelectric, piezoresistive [14,15], current and light, etc. [16]. Compared with other types of sensors, the force sensor based on optical fiber technology [17,18,19] has better performance in measurement accuracy, sensitivity and stability. In recent years, the research of polymer optical fibers has become popular. Polymer optical fibers not only have high flexibility, but also high sensitivity and a wide response range due to their low Young’s modulus [20]. There have been many developments in applications of FBGs inscribed in these fibers [21], but due to the high transmission loss of polymer optical fibers, polymer fiber grating technology is still in the research stage and has not been applied practically. At present, commercialized FBG sensors are still based on silica FBGs.

Various studies have been carried out to develop force sensors for microsurgery, micromanipulation, minimally invasive surgery, and other surgical disciplines. Fiber optic force sensors have been integrated into probes [22,23] or catheters [24,25,26] to obtain tactile information. Menciassi et al. [27] fabricated a robotic 17 × 7.5 × 0.4 mm micro-forceps using the Lithography, Electroplating, and Molding (LIGA) process. Strain gauges are incorporated as force sensors to measure the grip force. Bell et al. [28] developed a force sensing micro-forceps with fiber Bragg grating (FBG) for stapedectomy. The crimp force measurement can be used to increase the reproducibility of the crimp process. Zhang [29] incorporated a micrograting-based force sensor into a cell manipulation probe to measure the penetration force with microNewton resolution. Seibold et al. [30] designed a robotic MIS instrument with distal force sensing. The six-axis force torque sensor consists of six stain gauges mounted on a miniature Steward platform. Puangmali et al. [31] designed a triaxial distal force sensor for tissue palpation in minimally invasive surgery, also based on intensity modulated fiber optic sensor. Polygerinos et al. [32] developed a force sensor with FBG for cardiac surgery. Optical fiber sensors integrated at the end of surgical instruments in minimally invasive surgery were made by 3D printing [33]. Roesthuis et al. [34] integrated the FBG sensor into a flexible needle to obtain the axial strain at different positions on the flexible needle during the puncture process. Shang et al. [35] integrated the optical fiber sensor on the motor platform during puncture surgery. In the process of motor driving the puncture needle, the puncture force of the needle tip is obtained by measuring the deformation of the platform. Brett et al. [36] developed a variation pattern of force at the tip of the probe during the spinal puncture surgery. Berkelman et al. [37] developed a miniature triaxial force sensor based on strain gauges. Furthermore, various distal force sensing techniques for minimally invasive surgery applications have been developed based on fiber optical sensora [38]. Although the aforementioned works show promising results, most force sensors are designed to be mounted in the handle of a minimally invasive surgery instrument, and they cannot distinguish the contact force applied at the instrument tip and the contact force at the tissue. Furthermore, they are not able to sense the change of torque, but only sense axial force during puncture surgery.

Puncture surgery imposes strict dimensional constraints and high sensing performance requirements for sensorized instruments [39]. In order to directly measure the tool-to-tissue interaction force at the instrument tip, our approach is to incorporate fiber optic sensors [40] into the distal portion of the instrument shaft that is typically located inside the tissue. Our group has developed a new type of puncturing needle sensor based on FBG for human puncture surgery. The sensor can not only sense the change of axial force, but also sense the change of torque force. FBG strain sensors are not limited by the possible phase discontinuity and offer the advantages of fiber optic sensors with small dimension, high sensitivity, low cost, sterilization, biocompatibility, and immune from electrostatic and electromagnetic noise. Therefore, we designed a new type of puncturing needle sensor. Herein, the design, fabrication, calibration, and experiment results will be described and discussed. In this paper, the purpose is to solve the poor tactile sensing problem during human puncture surgery, so that doctors can better sense the change of axial force and the torque, to achieve the purpose of improving the accuracy, stability, quality, and reducing the potential damage of patients.

## 2. Structure Design of New-Type Puncturing Needle

**Figure 1:**
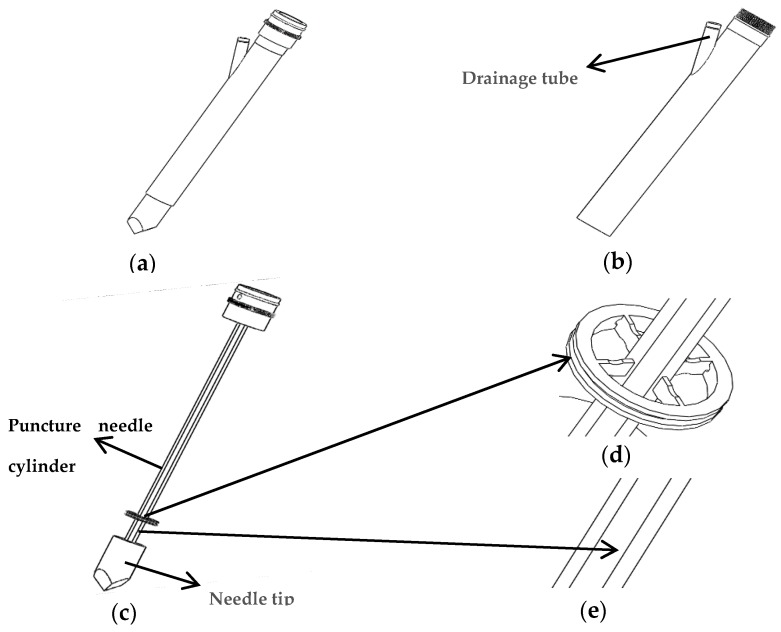
The new type of puncturing needle sensor: (**a**) Overall structure of the puncturing needle; (**b**) External sleeve of the puncture needle; (**c**) Internal structure of the puncture needle; (**d**) Spoke-type structure; (**e**) Groove on the surface of the puncture needle cylinder.

The structure of the new type of puncturing needle for human puncture surgery is presented in Figure 1a. It is mainly composed of an external sleeve and an internal puncture needle. Figure 1b illustrates the structure of the external sleeve, which has a drainage tube. Figure 1c illustrates the internal puncture needle cylinder, which is mainly used for human tissue puncture. A spoke-type structure is designed near the needle tip. A groove structure is dug on the surface of the puncture needle cylinder, as presented in Figure 1d,e.

**Figure 2:**
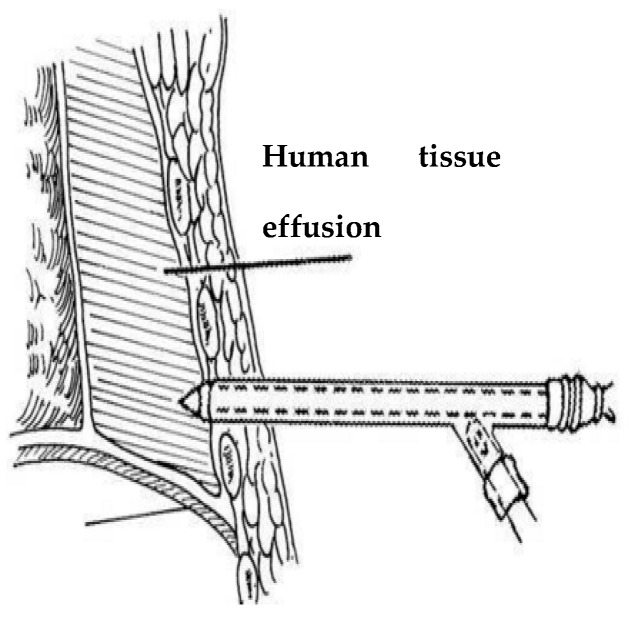
Diagram of thoracentesis and drainage.

**Figure 3:**
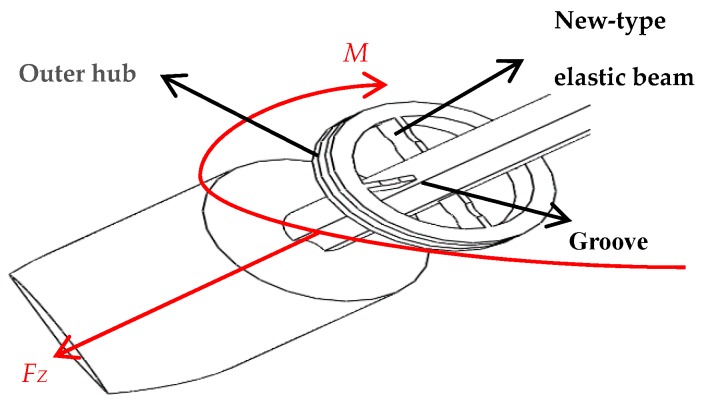
Force analysis of axial force FZ and torque M.

Extraction of effusions is one of the most important tasks during puncture surgery, and axial force and torque force are the main tactile forces sensed during puncture surgery, as presented in Figure 2. Because the puncture needle is mainly subjected to an axial force and a torque force during the human puncture surgery, the stress analysis of the new type of puncturing needle sensor during the human puncture surgery, as presented in Figure 3. With the spoke-type structure fixed on the puncturing needle cylinder, a torque force M will be generated in the outer hub when the new type of puncturing needle sensor is subjected to a tiny torque force, which leads to an elastic deformation of new type of elastic beam. The first FBG1 sensor is pasted on the upper surface of the new type of elastic beam and can be used to calibrate the torque force by detecting the change of the Bragg wavelength. The second FBG2 sensor is pasted on the under surface of the new type of elastic beam to sense the change of temperature. The puncture needle cylinder connecting the needle tip will be deformed, when the needle tip is subjected to the axial force F_Z_. The third FBG3 sensor is pasted into the grooves on the surface of the puncture needle cylinder, and its Bragg wavelength also changes with the deformation. The strain of axial force can also be calibrated by the change of the Bragg wavelength.

## 3. Simulation and Implementation

### 3.1. Principle of Operation

When a tiny torque force M is applied on the outer hub, the FBG1 sensor generates an elastic strain due to the stress deformation on the new type of elastic beam, so the Bragg wavelength of the FBG1 sensor changes. In the same way, when the needle tip is subjected to the axial force *F_Z_*_,_ the puncture needle cylinder connecting the needle tip will be deformed. The Bragg wavelength of sensor FBG3 will be changed, which is expressed as:

(1) $$M=f(\Delta \lambda ),F_{Z}=f(\Delta \lambda_{})$$

where *M* denotes the torque force is applied to the new-type elastic beam, *F_Z_* denotes the axial force is applied to the puncture needle cylinder, and Δλ denotes the shift in Bragg wavelength.

The shift in Bragg wavelength of the FBG sensors is linearly dependent on local strain and temperature change:

(2) $$\frac{\Delta \lambda }{\lambda }=K_{T}\Delta T+K_{\varepsilon }\Delta \varepsilon$$

where Δε denotes the local strain, ΔT denotes the temperature change, $K_{\varepsilon }$ and $K_{T}$ are constant coefficients associated to strain and temperature, respectively.

The three groups of FBGs have the same constant coefficients $K_{\varepsilon }$ and $K_{T}$, because of the use of the same material of optical fiber. The temperature change is the same for the three FBG sensors during the puncture surgery. Reference sensor FBG2 is not affected by strain ε, and is only affected by temperature *T*:

(3) $$\mathrm{FBG}1:\;\frac{\Delta \lambda_{1}}{\lambda_{1}}=K_{T}\Delta T+K_{\varepsilon }\Delta \varepsilon ;\;\mathrm{FBG}2:\;\frac{\Delta \lambda_{2}}{\lambda_{2}}=K_{T}\Delta T$$

(4) $$\Delta \lambda_{1}=\lambda_{1}{}^{\prime }-\lambda_{1};\;\Delta \lambda_{2}=\lambda_{2}{}^{\prime }-\lambda_{2}$$

Combining Equations (3) and (4), using reference optical fiber method, we can obtain the following equation:

(5) $$\frac{\Delta \lambda_{1}}{\lambda_{1}}-\frac{\Delta \lambda_{2}}{\lambda_{2}}=\frac{\lambda_{1}{}^{\prime }-\lambda_{1}}{\lambda_{1}}-\frac{\lambda_{2}{}^{\prime }-\lambda_{2}}{\lambda_{2}}=\frac{\lambda_{1}{}^{\prime }-\lambda_{2}{}^{\prime }}{\lambda_{1}}=K_{\varepsilon }\Delta \varepsilon$$

where $\lambda_{1}{}^{\prime }$, $\lambda_{2}{}^{\prime }$ denote the final state Bragg wavelength of two FBGs, and $\lambda_{1}$, $\lambda_{2}$ denote the initial Bragg wavelength of two FBGs. The initial Bragg wavelength of the FBGs is similar, it is about 1550 nm. $\Delta \lambda_{1}$, $\Delta \lambda_{2}$ are much smaller than $\lambda_{1}$, $\lambda_{2}$, so $\lambda_{1}$ can be regarded as approximately equal to $\lambda_{2}$. The different Bragg wavelength of FBGs has little effect on the sensitivity. The influence of temperature can be eliminated by using this reference FBG method

### 3.2. Force Simulation Analysis

**Figure 4:**
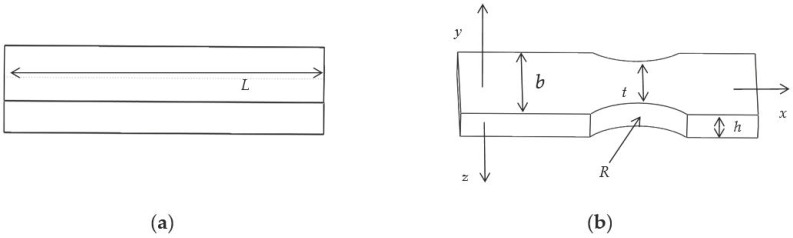
The structures of the elastic beams: (**a**) Traditional elastic beam; (**b**) new type of elastic beam.

The structure of the traditional elastic beam is presented in Figure 4a. The new type of puncturing needle sensor cuts the two ends of the traditional elastic beam into a new type of elastic beam, as presented in Figure 4b, where R is the radius of the cutting double-arc, and t is the minimum axial width of the new type of elastic beam, and b is the axial thickness, and h is the radial width.

The requirements for the new type of elastic beam are higher strength and less stiffness. After a certain amount of torque is applied, a micro-strain is generated at the grating pasting portion. The four new type of elastic beams are subjected to the same force, and the four new type of elastic beams have the same size. When a tiny torque force is applied to the new type of elastic beam, the linear displacement Δy of the new type of elastic beam along the y axis, is expressed as:

(6) $$\Delta y=\frac{9\pi R^{\frac{1}{2}}}{2E{\mathrm{ht}}^{\frac{5}{2}}}•\sqrt{R(b-t)-\frac{1}{4}(b-t{)}^{2}M}$$

From Equation (6), in order to improve the linear displacement Δy under the same amount of torque force, we can reduce *E*, h and t.

In general, the overall diameter of a puncture drainage needle is about 10 mm, and the diameter of a puncture needle cylinder is about 3 mm. In order to approach the actual operation of puncture drainage, it is more reasonable to design the length of traditional elastic beam as 3 mm. In this work, when the same torque is applied in the traditional elastic beam, finite element analysis of different axial thickness b and radial width h is also carried out, the results of simulation as presented in Table 1.

The results in Table 1 further support the previous analysis of the mechanical properties. The maximum stress intensity of a traditional elastic beam can be increased by adjusting the axial thickness b and radial width h. When the axial thickness b and radial width h are 1.4 mm and 0.5 mm, respectively, the elastic beam has better stress intensity.

**Figure 5:**
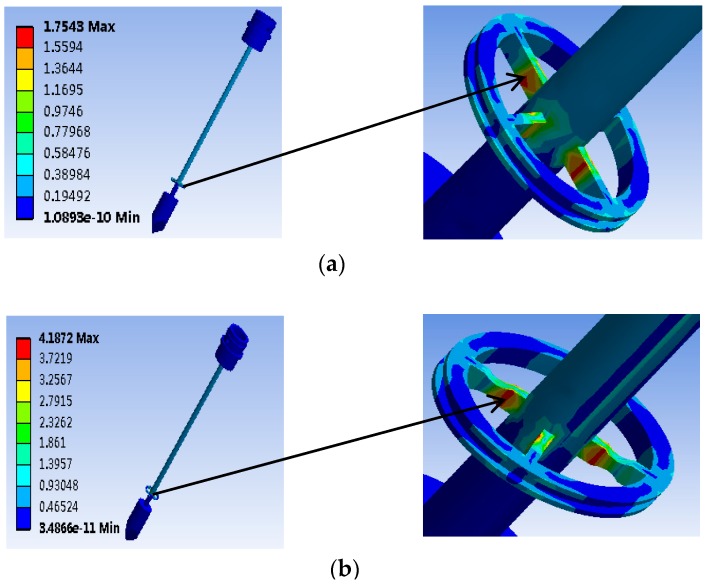
Simulation result: (**a**) Traditional elastic beam; (**b**) New type of elastic beam.

The finite element simulation of the traditional elastic beam and the new type of elastic beam are carried out, respectively to obtain the pressure cloud map (MPa). The simulation results are presented in Figure 5. When the same torque of 1 mN·m is applied in the elastic beam, the new type of elastic beam structure shows a significant increase in the maximum strain and strain sensitive area than the traditional elastic beam structure. Then we cut the two ends of the traditional elastic beam to form the new type of elastic beam. The minimum axial width of the new type of elastic beam t = 0.6 mm.

Combined with the finite element analysis results and size requirement of the puncture drainage needle, the size variables of the new type of elastic beam proposed in this paper are finally selected: the length L = 3 mm, the minimum axial width t = 0.6 mm, the axial thickness b = 1.4 mm, and the radial width h = 0.5 mm.

Simulation results show that the maximum strain of the new type of elastic beam is close to the that of puncture needle cylinder. Therefore, in order to obtain the largest deformation, the FBG1 sensor should be pasted to the upper surface of the new type of elastic beam which near the puncture needle cylinder.

**Figure 6:**
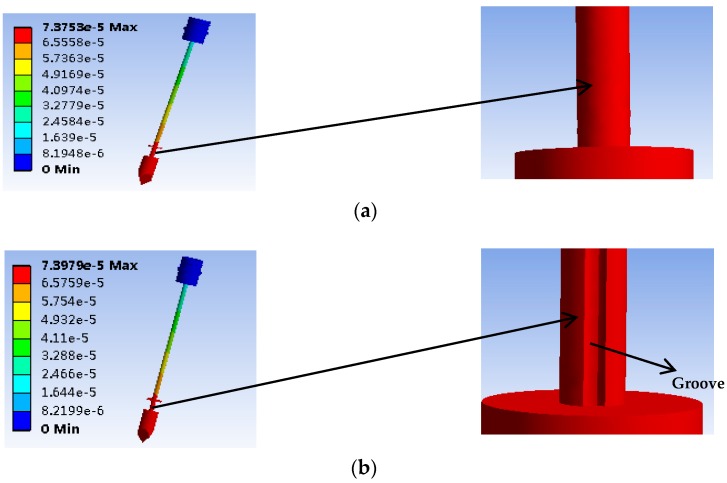
Simulation result: (**a**) Traditional puncture needle cylinder; (**b**) New type of puncture needle cylinder.

A finite element simulation is performed by applying the same 1N axial force to the new type of puncture needle cylinder and the traditional puncture needle cylinder, to obtain the displacement diagram (mm). The simulation results when the same axial force is applied to the needle tip, and the shape variables of the new type of puncture needle cylinder has been improved compared with the traditional puncture needle cylinder are presented in Figure 6. The maximum deformation on the new type of puncture needle cylinder is near the needle tip. The groove is convenient for the FBG sensor package. Therefore, the FBG3 sensor should be pasted into the groove of the puncture needle cylinder near the needle tip to obtain the largest axial force.

### 3.3. The FBG Sensor Paste

**Figure 7:**
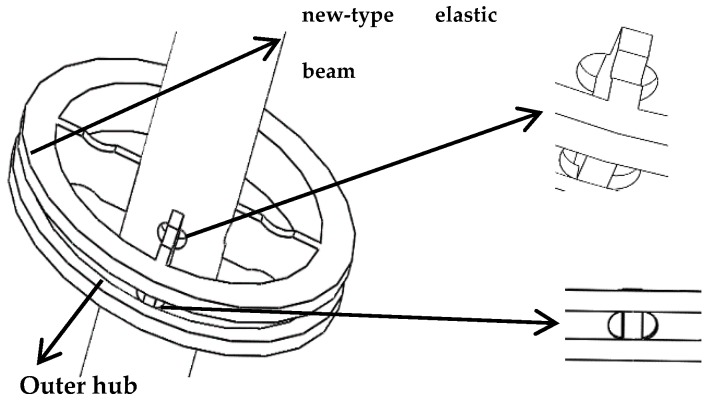
Small hole structure on the surface of outer hub and puncture needle cylinder.

**Figure 8:**
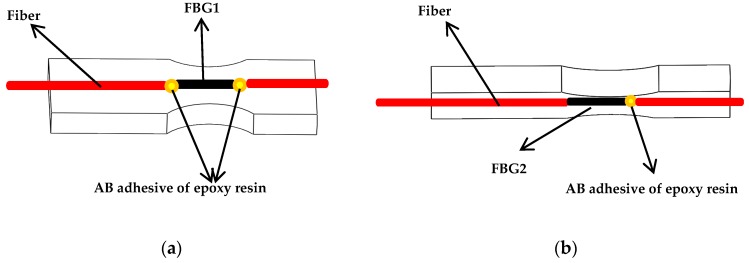
Two pasting methods of FBG1 and FBG2: (**a**) Two ends of FBG1 are pasted on the new type of elastic beam; (**b**) One end of FBG2 is pasted to the new type of elastic beam.

In order to get the maximum strain due to the torque force, the simulation results show that FBG1 sensor should be pasted to the upper surface of the new-type elastic beam close to the puncture needle cylinder, and the FBG1 sensor is used to measure torque forces. In order to avoid difficulties in the pasting process, such as limitations on the size of spoke-type structure, and the quite short length of the grating two pairs of holes are symmetrically punched on the surface of the outer hub and the puncture needle cylinder, and the two pairs of holes are close to the new type of elastic beam, as presented in Figure 7. The torque force FBG1 sensor and the FBG2reference sensor are pasted precisely through the holes. The two ends of the torque force FBG1 sensor grating is pasted on the surface of the new type of elastic beam, and the Bragg wavelength of the grating will shift with the strain of the new type of elastic beam. The grating of the reference FBG2 sensor is only pasted on one end, so the Bragg wavelength of the grating will not shift with the strain of the new type of elastic beam. The shift of the Bragg wavelength of the reference FBG2 sensor is only affected by temperature. The grating is pasted on the surface of the new type of elastic beam, as presented in Figure 8.

**Figure 9:**
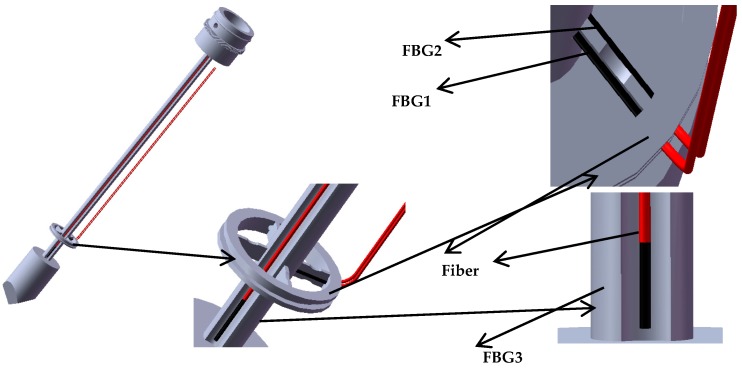
Three groups of FBGs paste position. FBG1 and FBG2 are pasted on the upper and lower surfaces of the new type of elastic beam, while FBG3 is pasted in groove on the surface of the puncture needle cylinder.

The simulation results show that the maximum deformation is near the needle tip when an axial force is applied to the new type of puncturing needle sensor, so the axial force FBG3 sensor should be pasted into a groove on the surface of the new type of puncture needle cylinder near the needle tip. In the experiment, three groups of FBG sensors are pasted with AB epoxy resin adhesive, because it has the advantages of high adhesive strength, small shrinkage, fast curing time and certain toughness. The pasting positions of the three groups of FBG sensors are presented in Figure 9.

## 4. Calibration and Experiments

### 4.1. Calibration with Experimental Data

**Figure 10:**
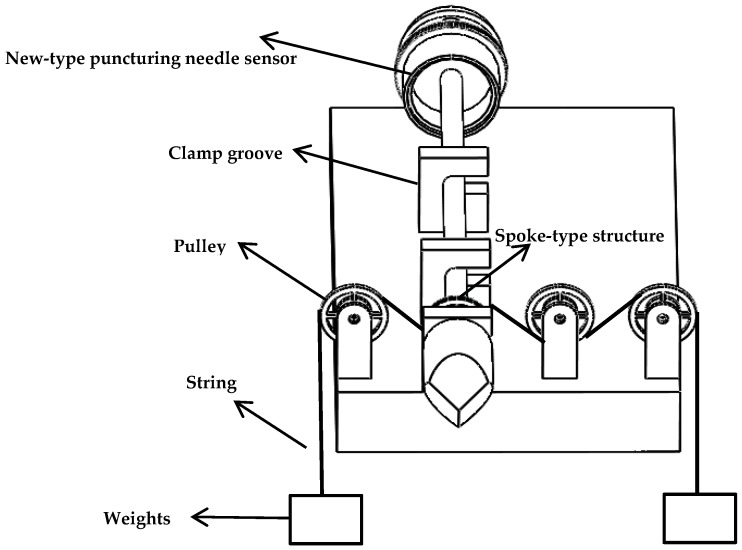
Structure diagram of the torque force experimental device.

According to the new type of puncturing needle sensor structure and size requirements, a new type of puncturing needle sensor is 3D printed for calibration experiments. In the torque force calibration experiment, Figure 10 illustrates the experimental device of the torque force sensing system. This experimental device is mainly composed of clamp groove, pulley, string, new-type puncturing needle sensor, weights and so on.

**Figure 11:**
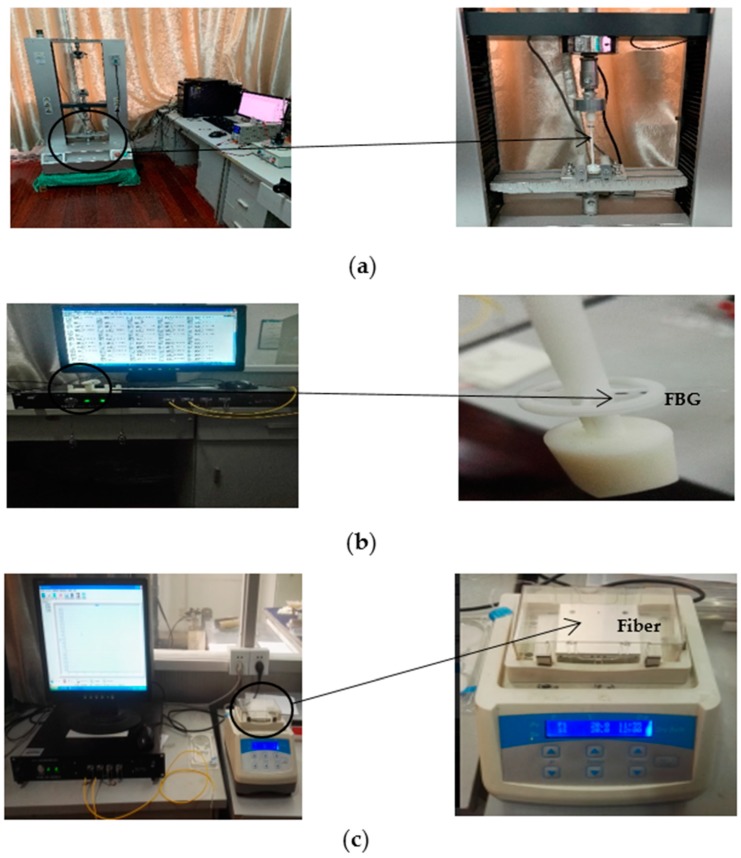
The experimental setup: (**a**) Axial force calibration experiments; (**b**) Torque force calibration experiments; (**c**) Temperature calibration experiments.

In the torque force experimental device, weights of equal weight are hung at both ends of the string. The outer hub produces a torque force due to the weight on the string, and the torsional force causes the elastic deformation of the new type of elastic beam, the magnitude of the torque force is changed by increasing or decreasing the mass of the weight. In this work, three groups of type I FBGs with grating length of 2 mm customized by the fiber manufacturer are selected, with reflectivity of more than 85%, bandwidth of less than 0.2 nm and side lobe suppression ratio greater than 10 dB. In the absence of torque force and axial force, a TGD-04 fiber Bragg grating high-speed intelligent demodulator is selected to detect the Bragg wavelength peaks of the three groups of FBG sensors. The instrument used for measuring the Bragg wavelength is very accurate. The 3-channel interface of the intelligent demodulator can detect the central wavelength of three groups of FBG sensors simultaneously. A CTM-2500 pressure calibration instrument is selected to detect the axial force. The temperature change of the FBGs is also calibrated. Figure 11 illustrates the experimental setup.

In the experiment, the torque force gradient is gradually increased in the positive stroke measurement, and then the torque force gradient is gradually reduced in the reverse stroke measurement. The axial force is calibrated by the same method. The Bragg wavelength values of the three groups of FBG sensors are measured by the fiber grating high-speed intelligent analyzer. There is a linear relationship between the tiny force and the difference of Bragg wavelength.

**Figure 12:**
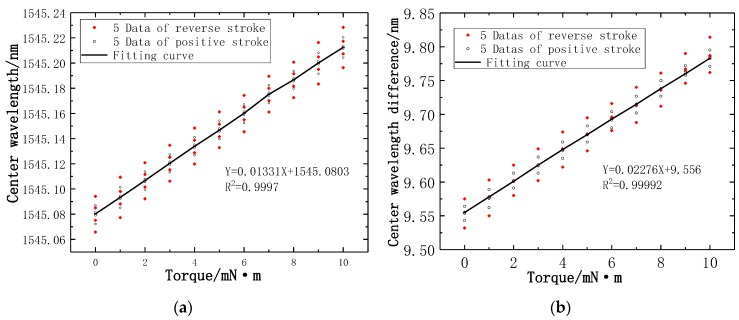
Wavelength and torque relation curves of two FBGs: (**a**) Center wavelength fitting curve of FBG1; (**b**) Fitting curve of central wavelength difference between FBG1 and FBG2.

In the torque force calibration experiment, when the torque force calibration range is 10 mN·m we recorded the multiple Bragg wavelengths at each corresponding torque force gradient. In a recent experiment, five datapoints are recorded at the torque gradient of positive and reverse stroke, respectively. A total of 10 data are recorded at each torque gradient. There is a linear relationship between the Bragg wavelengths measured by the torque force FBG1 sensor and the torque force gradient, the linear fitting as presented in Figure 12a. It can be seen from the slope of the fitting curve that the torque strain sensitivity is 13.3 pm/mN·m, and the determination coefficient R^2^ is 0.99977. Figure 12b illustrates the difference of central wavelength between the torque force FBG1 sensor and reference FBG2 sensor have a linear relationship with torque force gradient, which leads to that the fact the torque strain sensitivity is increased to 22.8 pm/mN·m, and the determination coefficient R^2^ is 0.99992.

**Figure 13:**
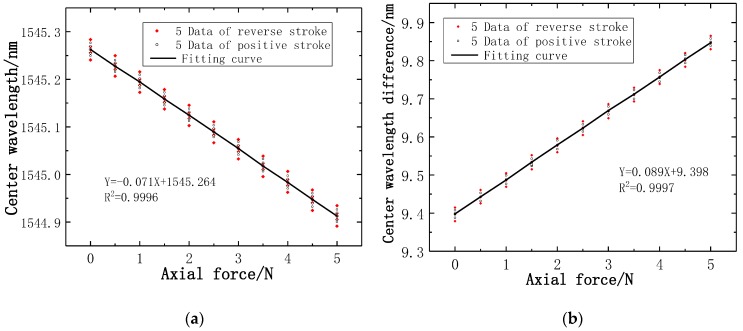
Wavelength and axial force relation curves of two FBGs: (**a**) Center wavelength fitting curve of FBG3; (**b**) Fitting curve of central wavelength difference between FBG3 and FBG2.

In the axial force calibration experiments, the axial force calibration range is 5 N. In the same way, five datapoints are recorded for the axial force gradient of positive and reverse stroke, respectively. A total of 10 data are recorded at each axial force gradient. There is a linear relationship between the Bragg wavelength measured by the axial force FBG3 sensor and the axial force gradient, as presented in Figure 13a. It can be seen from the slope of the fitting curve that the axial force strain sensitivity is 0.071 nm/N and the determination coefficient R^2^ of the FBG3 sensor is 0.9996. Figure 13b illustrates the difference of Bragg wavelength between axial force FBG3 sensor and reference FBG2 sensor has a linear relationship with axial force gradient, which leads to that the axial force strain sensitivity is increased to 0.089 nm/N, and the determination coefficient R^2^ is 0.9997.

**Figure 14:**
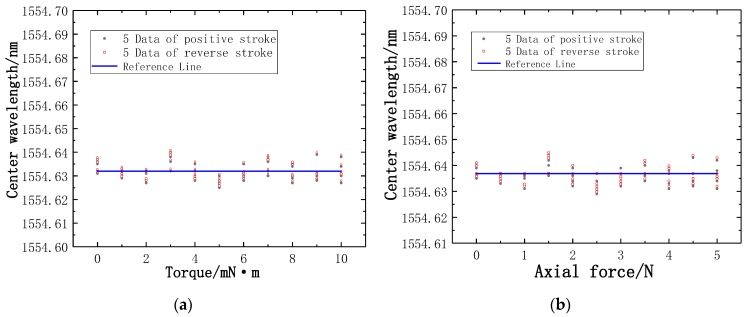
The trend of the values measured by FBG2: (**a**) The trend of the values measured by FBG2 in the torque test; (**b**) the trend of the values measured by FBG2 in the axial force test.

In the experiment, the main sensing fibers are FBG1 and FBG3, FBG2 is a reference fiber. FBG2 is not affected by the pressure and torque force. The little change of temperature is the main factor affecting the Bragg wavelength of FBG2, so the trend of the values measured by FBG2 is not obvious. The Bragg wavelength of FBG2 fluctuates slightly up and down near the reference line, as presented in Figure 14.

From the curve fitting diagram, the strain sensitivity of the new type of puncturing needle sensor is significantly improved by using the reference fiber method. The Bragg wavelength difference has the effect of temperature compensation. By reducing the impact of temperature on the measurement, the measurement accuracy is much higher.

**Figure 15:**
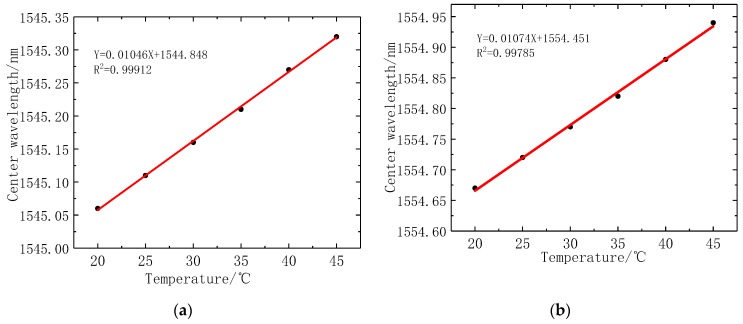

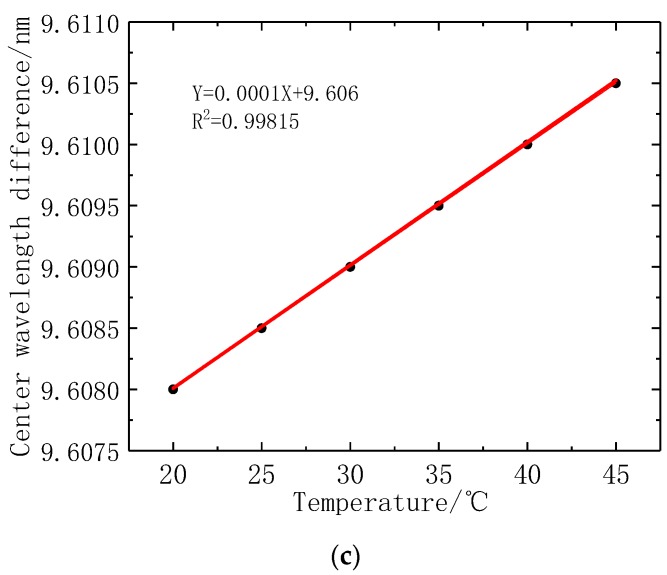
Wavelength and temperature relation curves of two FBGs: (**a**) Center wavelength fitting curve of FBG1; (**b**) Center wavelength fitting curve of FBG2. (**c**) Fitting curve of central wavelength difference between FBG1 and FBG2.

In this work, the temperature sensitivity coefficient of the fiber is calibrated. The temperature range is 20 °C~45 °C without any stress. The highest temperature of the human body is about 40 °C, so the temperature range of FBGs is limited to 45 °C. The Bragg wavelength of the FBG1 sensor and FBG2 sensor are recorded when the change of temperature gradient is 5 °C. Figure 15 illustrates the fitting curve of fiber grating temperature, where the FBG1 temperature coefficient is 10.5 pm/°C, and the temperature coefficient of FBG2 is 10.7 pm/°C. When using the reference fiber method for temperature compensation, the difference between FBG1 and FBG2 Bragg wavelength, is used as a measurement parameter. The optical fiber grating temperature coefficient decreased to 0.1 pm/°C. It shows that the reference fiber method has a good temperature compensation effect, and greatly reduces the influence of temperature change on stress measurement. It is further proved that the reference fiber method can improve the sensitivity of stress measurement and eliminate the influence of temperature changes.

### 4.2. Experiment and Analysis

The standard deviation is calculated from Bessel’s formula, which is expressed as:

(7) $$\sigma =\sqrt{\frac{\sum_{i-1}^{n}{\left(Y_{i}-\bar{Y}\right)}^{2}}{n-1}}$$

where σ denotes the standard deviation, n denotes the number of tests, $Y_{i}$ denotes the Bragg wavelength of tests, $\bar{Y}$ denotes the arithmetic mean of Bragg wavelength.

The experimental data of torque force and axial force are analyzed, 10 datapoints with different torque force or axial force gradient are taken as sample data. Table 2 illustrates the experimental standard deviation of the new type of puncturing needle sensor at different torque gradients in positive and reverse stroke, which is calculated by using the Bessel formula method. The Bragg wavelength difference between FBG1 sensor and FBG2 sensor at different torque force gradients are taken as the output date, and the arithmetic mean value of 10 groups of output date at the torque force gradient in the positive stroke or reverse stroke are taken as the average calibration point, and the difference between the average calibration points of positive and reverse stroke is the deviation value of torque force gradient, as presented in Table 3.

In the same way, Table 4 illustrates the experimental standard deviations at different axial force gradients in tests. Table 5 illustrates the average calibration point and deviation value of positive and reverse stroke in the axial force calibration experiment.

### 4.3. Performance of New-Type Sensors

**Figure 16:**
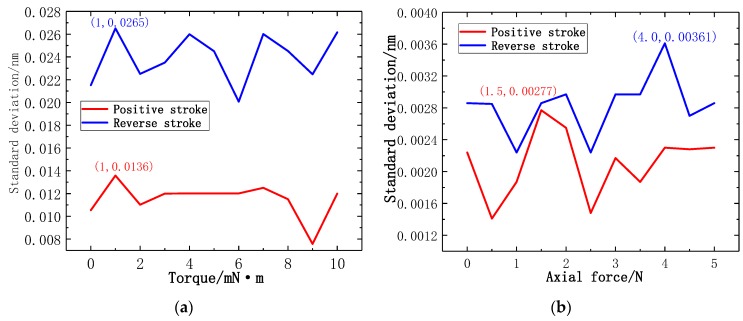
Line chart of standard deviation: (**a**) Standard deviation of torque; (**b**) Standard deviation of axial force.

The calculation of repeatability is expressed by the ratio of double or triple of the maximum standard deviation in positive and reverse stroke to full range, which is expressed as:

(8) $$Y_{R}=\pm \frac{2\sigma \sim 3\sigma }{Y_{F•S}}\times 100\%$$

where σ denotes the standard deviation, $Y_{R}$ denotes the repeatability, $Y_{F•S}$ denotes the full range. Figure 16a illustrates the line chart of the experimental standard deviation at different torque force gradients. In the calibration experiment of torque force gradient, the maximum experimental standard deviation is generated at 1 mN·m in reverse stroke. According to the maximum experimental standard deviation, the repetition error of the torque force test can be calculated as Y_R_ = 0.81%FS. Figure 16b illustrates the line chart of the experimental standard deviation at different axial force gradients. In the calibration experiment of axial force gradient, the maximum experimental standard deviation is generated at 4 N in reverse stroke. According to the maximum experimental standard deviation, the repetition error of the axial force test of the new-type puncturing needle sensor can be calculated as Y_R_ = 0.11%FS. The results show that the new-type puncturing needle sensor has good repeatability.

**Figure 17:**
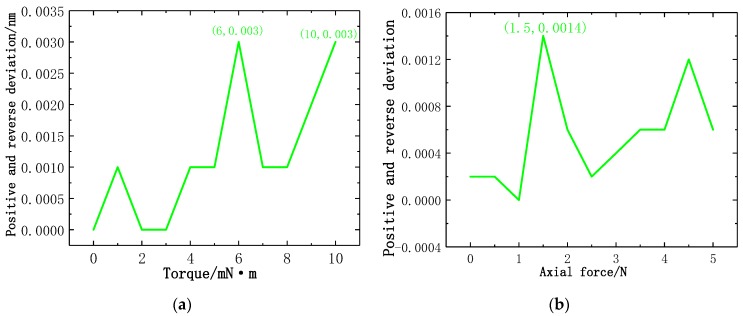
Line chart of positive and reverse deviation: (**a**) Positive and reverse deviation of torque; (**b**) Positive and reverse deviation of axial force.

The calculation of hysteresis is expressed by the ratio of the maximum deviation or half of the maximum deviation in the positive and reverse stroke to the full range, which is expressed as:

(9) $$Y_{H}=\pm \frac{\Delta H_{\max}}{Y_{F•S}}\times 100\%$$

(10) $$Y_{H}=\pm \frac{\Delta H_{\max}}{2Y_{F•S}}\times 100\%$$

where $\Delta H_{\max}$ denotes the maximum deviation of the average calibration point in positive and reverse stroke, $Y_{H}$ denotes the hysteresis. Figure 17a illustrates the line chart of positive and reverse deviation at different torque force gradients. In the calibration experiment of torque force gradient, the maximum positive and reverse deviation is generated at 6 mN·m and 10 mN·m. According to the maximum positive and reverse deviation, the hysteresis error of the torque force of the new type of puncturing needle sensor is Y_H_ = 0.03%FS. Figure 17b illustrates the line chart of positive and reverse deviation at different axial force gradients, the maximum positive and reverse deviation is generated at 1.5 N. According to the maximum positive and reverse deviation, the hysteresis error of the axial force of the new type of puncturing needle sensor is Y_H_ = 0.014%FS. The results show that the new type of puncturing needle sensor has good hysteresis.

**Figure 18:**
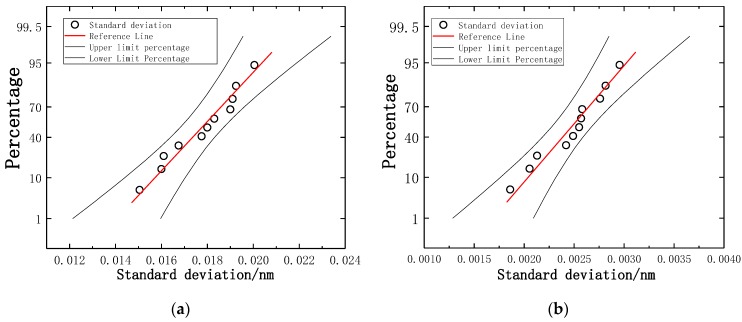
Probability distribution of standard deviation: (**a**) Probability distribution of torque; (**b**) Probability distribution of axial force.

The calculation of resolution is expressed by ±σ of the standard deviation of multiple measurements. The arithmetic mean of each standard deviation is used as a reference point. Figure 18a illustrates the probability distribution of torque standard deviation. Figure 18b illustrates the probability distribution of axial force standard deviation. According to the probability distribution and sensitivity, torque resolution is 0.8 mN·m and axial force resolution is 0.03 N.

### 4.4. Transmission Spectra of the FBGs

**Figure 19:**
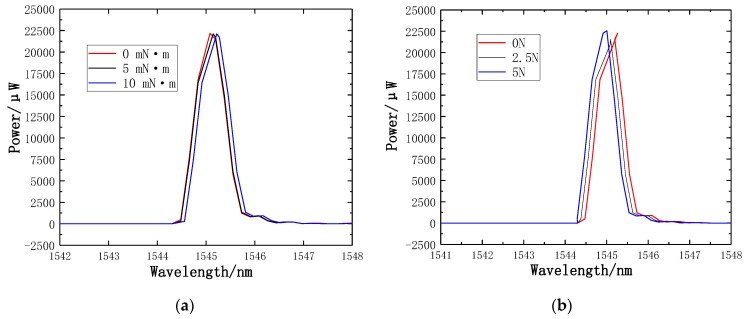
Transmission spectra of the FBGs: (**a**) Transmission spectra of torque; (**b**) Transmission spectra of axial force.

The TGD-04 FBG demodulator, which has the advantages of being a powerful acquisition system, with high reliability and fast data acquisition speed, is used to record transmission spectra and the wavelength shifts of FBGs. Figure 19a illustrates the transmission spectra of torque 0 mN·m, 5 mN·m and 10 mN·m, respectively. Figure 19b illustrates the transmission spectra of axial force 0 N, 2.5 N, and 5 N, respectively.

## 5. Discussion

The results have shown that the proposed new type of puncturing needle sensor can reliably monitor both axial force and torque force simultaneously using a new type of structure. The FBG sensing method uses the Bragg wavelength difference to calibrate the axial force and torque force, which make the sensing more sensitive. One of the advantages of the new type of puncturing needle sensors is the special structure. A spoke-type structure is designed near the needle tip. The FBG1 sensor and the reference FBG2 sensor are pasted on the upper and lower surfaces of the new type of elastic beam, and the FBG3 sensor is pasted into the groove on the surface of the new type of puncturing needle cylinder. The sensitivity increases with the new type of structure. The design of the new type of puncturing needle sensor is extremely compact because the grating is pasted in new type of elastic beam having a length < 3 mm. To avoid the difficulty of pasting, two pairs of holes are symmetrically punched on the surface of the outer hub and the puncture needle cylinder, and the two pairs of holes are close to the new type of elastic beam, where holes facilitate grating pasting. The new type of structure shows a significant increase in the maximum strain and strain sensitivity. The new type of sensor provides reliable repeatability, good hysteresis and higher resolution.

Compared to the results achieved by other studies, our sensory system could obtain both axial force and tiny torque forces simultaneously during the puncture process. Berkelman et al. [37] developed a miniature triaxial force sensor based on strain gauges, but it is designed to be mounted in the handle of a microsurgical instrument. A handle-mounted force sensor is not practical for minimally invasive surgery, because it cannot distinguish between the force applied at the instrument tip and the contact force at the sclera [41]. The study reported in [42] developed a triaxial force sensing laparoscopic instrument with a diameter of 5 mm for force feedback during minimally invasive robotic surgery (MIRS). The force sensor is based on intensity modulated fiber optic sensors and can provide a resolution of 0.04 N. A similar article was presented by Puangmali et al. [31] focusing on palpation by means of miniature 3-axis distal force sensor in MIS, and can provide a resolution of 0.02 N. A similar study in [32] developed a triaxial catheter-tip force sensor for MRI-guided cardiac procedures, and can provide a resolution of 0.02 N. The sensor has good repeatability and hysteresis. He et al. [22] developed a sub-millimetric 3-DOF force sensing instrument for retinal microsurgery. The axial force resolution of the sensor is below 1 mN while the transverse force resolution is less than a 0.25 mN. Because it is suitable for retinal microsurgery, the requirement for resolution of the sensor is very high, but it still has no ability to measure torque. Usually, the force in punctures ranges from 0.2 N to 4 N. These are the forces required to puncture the tissue. This paper developed a new type of puncturing needle sensor structure for puncture and drainage in MIS. The sensor not only guarantees a high axial force resolution, but also has the ability to detect the torque force. Meanwhile, the sensor has better repeatability and hysteresis. A table comparing our results with the corresponding data provided by previous works for similar systems as reported by other authors, as presented in Table 6.

## 6. Conclusions

This paper presents a new type of puncturing needle sensor based on fiber Bragg gratings which can measure axial force and torque force. A new type of elastic beam structure is designed near the needle tip of the new type of puncture needle, and the upper and lower surfaces of the new type of elastic beam are used to paste the FBG1 sensor and the reference FBG2 sensor. Both ends of the grating of the FBG1 sensor are pasted on the upper surface of the new type of elastic beam, while only one end of the grating of the reference FBG2 sensor is pasted on the lower surface of the new type of elastic beam, and a grooved part is engraved on the surface of the puncture needle cylinder to paste the FBG3 sensor. FBG1 sensor is used to detect the change of torque force, while the FBG3 sensor is used to detect the change of axial force. Using the reference fiber method eliminates the influence of temperature. The simulation analysis and sensing experiment show that when the torque force calibration is in the range of 10 mN·m, the torque sensitivity of the new type of FBG sensor is 22.8 pm/mN·m, and the determination coefficient R^2^ is 0.99992, and the hysteresis error is 0.03%FS, and the repetition error is 0.81%FS. When the axial force calibration range is 5 N, the axial force sensitivity of the new type of FBG sensor is 0.089 nm/N, the determination coefficient R^2^ is 0.9997, and the hysteresis error is 0.014%FS, and the repetition error is 0.11%FS. The axial force resolution and torque resolution of the new type of puncturing needle sensor are 0.03 N and 0.8 mN·m, respectively. The advantage of the new type of puncturing needle sensor based on fiber Bragg gratingd is that it can sense the strain of torque force and axial force, thus avoiding unnecessary collisions between surgical instruments and human organs during human puncturing surgery, and it can enhance the ability of doctors to sense tiny force changes, so this method also enhances the safety of minimally invasive surgery and has high practicality and versatility.

## Figures and Tables

**Table 1:** The finite element analysis results of different traditional elastic beam size.

Number	Length of Elastic Beam L/mm	Radial Width h/mm	Axial Thickness b/mm	Maximum Stress Intensity/MPa
1	3	0.5	1.4	8.77
2	3	0.5	1.6	6.04
3	3	0.5	1.8	4.67
4	3	0.6	1.4	4.45
5	3	0.6	1.6	3.16
6	3	0.6	1.8	2.12

**Table 2:** Standard deviation of each torque gradient in the same stroke.

Torque/mN·m	Standard Deviation of Positive Stroke/nm	Standard Deviation of Reverse Stroke/nm
0	0.0105	0.0215
1	0.0136	0.0265
2	0.0110	0.0225
3	0.0120	0.0235
4	0.0120	0.0260
5	0.0121	0.0245
6	0.0121	0.0201
7	0.0125	0.0260
8	0.0115	0.0245
9	0.0076	0.0225
10	0.0120	0.0262

**Table 3:** The average calibration points and deviation values of torque gradient.

Torque/mN·m	Positive Stroke Average Calibration Point/nm	Reverse Stroke Average Calibration Point/nm	Positive and Reverse Deviation/nm
0	9.554	9.554	0.000
1	9.576	9.577	0.001
2	9.602	9.602	0.000
3	9.625	9.625	0.000
4	9.647	9.648	0.001
5	9.671	9.670	0.001
6	9.692	9.695	0.003
7	9.715	9.714	0.001
8	9.738	9.737	0.001
9	9.763	9.765	0.002
10	9.783	9.786	0.003

**Table 4:** Standard deviation of each axial force gradient in the same stroke.

Axial Force/N	Standard Deviation of Positive Stroke/nm	Standard Deviation of Reverse Stroke/nm
0.0	0.00224	0.00286
0.5	0.00141	0.00285
1.0	0.00187	0.00224
1.5	0.00277	0.00286
2.0	0.00255	0.00297
2.5	0.00148	0.00224
3.0	0.00217	0.00297
3.5	0.00187	0.00297
4.0	0.00230	0.00361
4.5	0.00228	0.00270
5.0	0.00230	0.00286

**Table 5:** The average calibration points and deviation values of axial force gradient.

Axial Force/N	Positive Stroke Average Calibration Point/nm	Reverse Stroke Average Calibration Point/nm	Positive and Reverse Deviation/nm
0.0	9.3980	9.3978	0.0002
0.5	9.4430	9.4428	0.0002
1.0	9.4870	9.4870	0.0000
1.5	9.5352	9.5338	0.0014
2.0	9.5790	9.5784	0.0006
2.5	9.6228	9.6230	0.0002
3.0	9.6688	9.6684	0.0004
3.5	9.7120	9.7114	0.0006
4.0	9.7564	9.7570	0.0006
4.5	9.8038	9.8026	0.0012
5.0	9.8474	9.8468	0.0006

**Table 6:** Comparative table with the corresponding data.

Sensor Type	Uses	Directivity	Sensitivity	Repeatability Error and Hysteresis Error	Resolution
New type of puncturing needle sensor	Puncture and drainage in MIS	Axial force and torque	0.089 nm/N 22.8 pm/mN·m	Torque: Y_R_ = 0.81%FS, Y_H_ = 0.03%FS; Axial: Y_R_ = 0.11%FS, Y_H_ = 0.014%FS	Torque: 0.8 mN·m; Axial: 0.03 N
5 mm Diameter tri-axial force sensor [42]	Force feedback during MIRS	Tri-axial	10.3 mV/μm	No mention	0.04 N
Miniature 3-Axis Distal Force Sensor [31]	MIS Palpation	3-Axis	No mention	Hysteresis = 3.5%.	0.02 N
Triaxial Catheter-Tip Force Sensor [32]	MRI-Guided Cardiac Procedures	Triaxial	0.5V/N	Repeatability > 95% Hysteresis ratio = 5.3%	0.01N
DOF Force Sensing Instrument [22]	Retinal Microsurgery	3-DOF	No mention	Repeatability = 1.3 pm	Axial:1 mN; Transverse: 0.25 mN
